# The characteristics and long-term survival of patients with colorectal liver metastases with pathological complete response after chemotherapy

**DOI:** 10.7150/jca.47911

**Published:** 2020-08-27

**Authors:** Da Xu, Xiao-Luan Yan, Jia-Ming Liu, Juan Li, Bao-Cai Xing

**Affiliations:** Key laboratory of Carcinogenesis and Translational Research (Ministry of Education/Beijing), Hepatopancreatobiliary Surgery Department I, Peking University Cancer Hospital & Institute, Beijing 100142, China.

**Keywords:** Colorectal neoplasms, liver neoplasms, chemotherapy, pathological complete response, surgery

## Abstract

**Purpose:** Preoperative chemotherapy is widely used for colorectal liver metastasis (CRLM). Pathological complete response (PCR) after chemotherapy indicates complete tumor regression and an extremely favorable prognosis. This study aimed to explore the characteristics and long-term survival of CRLM patients with pCR, who underwent surgery after preoperative chemotherapy.

**Methods:** We retrospectively analyzed the clinical data of 494 CRLM patients who underwent hepatectomy after preoperative chemotherapy between January 2006 and January 2019. pCR was defined as the absence of any cancer cells on pathological examination.

**Results:** Thirty (6.07%) patients achieved pCR after preoperative chemotherapy; 70% patients who achieved pCR did not experience recurrence and were cured after hepatectomy. The long-term prognosis of patients with pCR was extremely favorable, with 10-year overall and disease-free survivals of 85.2% and 73.7%, respectively; these were significantly better than those of patients without pCR (31.3% and 15.2%, respectively). Liver metastases <3 cm, preoperative carcinoembryonic antigen level ≤20 ng/mL, primary T stage 1-2, and right-sided primary tumors were independent predictors for pCR.

**Conclusion:** pCR occurred in 6% of patients with CRLM after preoperative chemotherapy. Patients with a smaller tumor burden are more likely to benefit from chemotherapy and achieve pCR.

## Introduction

Surgical resection is currently the most effective treatment method for colorectal liver metastasis (CRLM), with a 5-year survival rate of 40-50% 1. Preoperative chemotherapy has been widely used in CRLM to reduce the tumor size and raise the curative resection rate; it has also been used to control microsatellite metastases and to allow for testing of the biological behavior of the tumor by assessing treatment response 2-4. With the development of chemotherapy and targeted drugs, the response rates to preoperative treatment have increased; some patients show clinical complete response (CCR), which is defined as lesion disappearance on imaging 5. However, CCR is not equivalent to true complete response, since approximately 30-70% of tumors that achieve CCR relapse during follow-up 6, 7. Pathological complete response (pCR), which refers to the lack of residual cancer cells in the tumor tissue, is determined based on careful pathological examination. Thus, pCR could reflect an extremely positive response to chemotherapy, more precisely than CCR 8.

pCR provides strong prognostic information in patients with CRLM who undergo hepatectomy after chemotherapy. A previous study found that approximately 4% of patients with CRLM achieve pCR after preoperative chemotherapy 8, 9. Further, these patients have extremely favorable long-term prognosis (10-year survival of 70%) 8. However, pCR can only be confirmed by pathological examination and is difficult to ascertain before surgery without resected specimens.

Some studies have tried to identify clinical factors associated with pCR after chemotherapy, but most of these studies were performed before 2010 when targeted therapy and gene status testing were not widely performed 6, 8. The data concerning predictive factors, survival, and pCR rate may have changed in recent years. This study aimed to explore the characteristics and long-term prognosis of CRLM patients with pCR, who underwent surgery after preoperative chemotherapy.

## Materials and Methods

All study participants provided written consent. The study design was approved by the Ethical Review Board committee of the Beijing Cancer Hospital and Institute (Beijing, China).

### Patient selection

Patients with CRLM who underwent liver resection after preoperative chemotherapy in the HPB Surgery Ward I at the Beijing Cancer Hospital between January 2006 and January 2019 were identified from our CRLM database. Some patients had their primary tumor resected at another institution, but all liver metastases were resected at our center. The patient exclusion criteria included the following: (1) non-radical surgery (R2 resection), (2) no information on pathological response, (3) unresectable extrahepatic metastases, and (4) loss to follow-up. Only the first surgery was included for patients who underwent multiple liver resections for recurrence.

### Pathologic response examination

**Figure 1:**
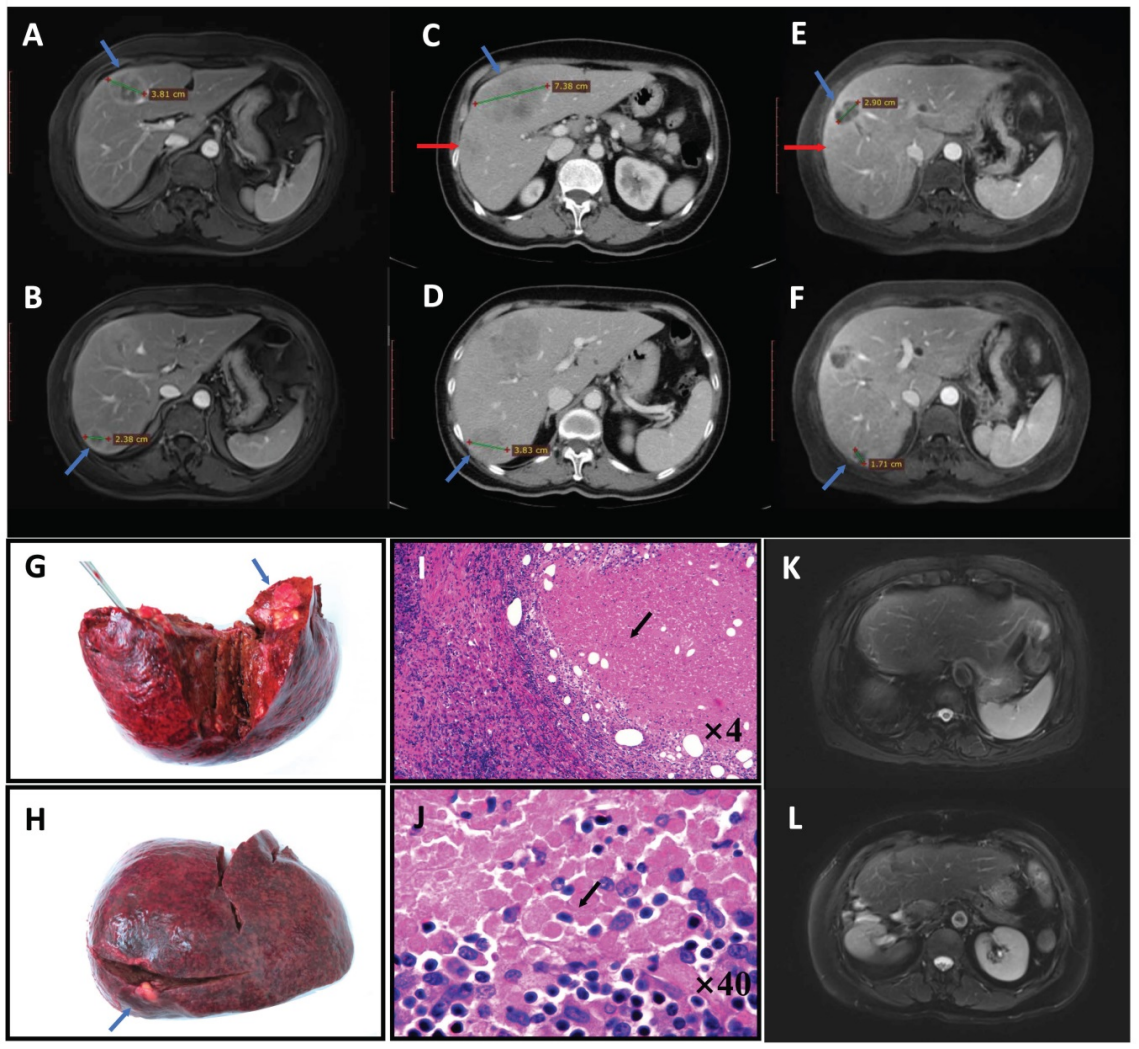
Pathological complete response (pCR) in a patient with colorectal liver metastases who received pembrolizumab. A 71-year-old woman was diagnosed with simultaneous liver metastases from ascending colon cancer in our center in August 2017. Gene testing showed the tumor was of the *RAS/BRAF* wild-type, MSI-H. MRI indicated two lesions (blue arrow) that were resectable (**A, B**). After two cycles of pembrolizumab, the tumor progressed and a new lesion appeared on CT (red arrow) (**C, D**). We administered two cycles of HAI with systematic oxaliplatin combined with two cycles of pembrolizumab, after which the tumor had significantly shrunk on MRI (**E, F**). Right hepatic lobectomy and simultaneous right hemicolectomy were then performed (liver specimen **G, H**). Pathologic examination found no tumor cells in either the liver or primary tumor tissue, with these regions having been replaced by tumor necrosis, fibro-collagenous proliferation and inflammatory cells (hematoxylin-eosin-stained, **I:** ×4 tumor-normal liver interface, **J:** ×40 tumor tissue), except for in one positive lymph node (No. 14v) (ypT0N1aM0). The patient was still disease-free after 2 years on MRI (**K, L**) and had all the four factors predictive of pCR in this study (liver metastases <3 cm, preoperative carcinoembryonic antigen level ≤20 ng/mL, primary T stage 1-2, and a right-sided primary tumor). Abbreviations: MSI-H, microsatellite instability-high; MRI, magnetic resonance imaging; CT, computed tomography; HAI, hepatic arterial infusion

All the tumors in each CRLM patient were sampled. The hematoxylin-eosin-stained sections were reviewed by two gastrointestinal pathologists. The pathologic response to chemotherapy was categorized based on the MD Anderson category 10. The area of residual viable tumor cells within each tumor was estimated as a percentage of the total tumor surface; pCR was defined as the absence of any cancer cells (**Figure 1I, J**). Tumors with pCR were submitted entirely for microscopic examination. Major and minor responses were defined as 1%-49% and ≥50% residual cancer cells, respectively. Patients with major and minor responses were all classified into the non-pCR group in this study.

### Preoperative management and liver resection

Preoperative chemotherapy with standard oxaliplatin- or irinotecan-based regimens was administered with or without targeted agents (bevacizumab and cetuximab) to patients with a high clinical risk score (CRS) or initial unresectable liver metastases. Anti-programmed cell death protein 1 (anti-PD-1) therapy was administered to patients with MSI-H status. Hepatic arterial infusion (HAI) was performed in patients whose metastases were still unresectable after several lines of systematic chemotherapy. The detailed protocol included a HAI bolus of oxaliplatin, leucovorin, and 5-fluorouracil (5-FU) for over 48 hours. The protocol was repeated every 1 month, with or without systemic targeted drug therapy. Gadoxetic acid/contrast-enhanced magnetic resonance imaging (MRI), chest computed tomography (CT), and pelvic CT/MRI scans were performed every 2-4 cycles during preoperative chemotherapy. Chemotherapy response was evaluated according to the Response Evaluation Criteria in Solid Tumours, version 1.1 11. Gene status was detected in every patient, including that of *KRAS*, *NRAS,* and BRAF V600E. The time interval between the last cycle of chemotherapy and liver surgery was usually 2-4 weeks and extended to 6-8 weeks in patients receiving bevacizumab. Primary tumors located in the cecum, ascending colon, and transverse colon were defined as right-sided tumors, and tumors in the splenic flexure, descending colon, sigmoid colon, and rectum were defined as left-sided tumors. Intra-operative ultrasonography was routinely performed to detect any lesions that had disappeared on imaging to ensure that all the tumors were radically resected. New lesions found during surgery were also resected.

### Follow-up

Postoperative follow-up was recommended every 3 months after surgery in the first 2 years and every 6 months in years 3-5; this included CT or MRI and assessment of carcinoembryonic antigen (CEA) and CA19-9 levels. Patients who developed recurrence during follow-up underwent localized treatment, including surgical or ablation techniques or palliative chemotherapy.

### Statistical analyses

Categorical variables have been presented as numbers with percentages and were compared using the Chi-squared test. Continuous variables are described by ranges, and intergroup comparisons were performed using the t-test. Multivariable logistic regression analysis was performed to determine independent predictors of pCR. Disease-free survival (DFS) and overall survival (OS) were calculated from the date of hepatectomy to the date of recurrence and the date of death or last follow-up, respectively. Survival curves were plotted using the Kaplan-Meier method and were compared using the log-rank test. All statistical analyses were conducted using SPSS software version 24.0 (IBM Corp., Armonk, NY, USA). A *P* < 0.05 was considered statistically significant.

## Results

### Characteristics and preoperative treatment details of patients

A total of 494 patients were enrolled in this study. The median follow-up duration was 24 months, and the median age of the cohort was 55 (33-77) years. The baseline characteristics of the patients are summarized in **Table 1.** Thirty (6.07%) patients, including 13 women and 17 men achieved pCR on pathological examination (pCR group). Patients with pCR had smaller liver metastases (*P* < 0.001), lower preoperative CEA (*P* = 0.001), and earlier primary T stage (*P* = 0.034). A higher proportion of patients with pCR showed complete or partial response on imaging compared with patients who did not achieve pCR; however, this was not significant (56.7% vs 45.3%, *P* = 0.224).

The details of chemotherapy have been presented in **Table 2.** The median number of chemotherapy cycles in both, the pCR group (range 2-10) and non-PCR group (range 1-25) were 4, with no statistical difference; 276 (55.9%) patients received targeted drugs combined with chemotherapy, including 162 (32.8%) and 114 (23.1%) who had received bevacizumab and cetuximab, respectively. There was no difference between the pCR and non-pCR groups in terms of the percentages of patients receiving cetuximab (*P* = 0.390) or bevacizumab (*P* = 0.737). The proportion of patients who received systemic chemotherapy with oxaliplatin was slightly higher in the pCR than in the non-PCR group (76.7% vs 62.5%), but there was no statistical difference (*P* = 0.119). However, only 20% pCR patients received an irinotecan-based regimen; this was significantly lower than the proportion in non-pCR patients (40.7%) (*P* = 0.024).

### Treatment scheme of different time periods

Using 2012 as the boundary, we analyzed the proportion of pCR that occurred in different time periods and the changes in specific preoperative treatment regimens (**Table 3**). The results showed that 13 (13/210) patients before 2012 and 17 (17/284) patients after 2012 showed pCR, and there was no significant difference (*P* = 0.93). However, in terms of the treatment scheme, 52.9% pCR patients received combined targeted therapy after 2012; although this was higher than 23.1% before 2012, there was no statistical difference. In addition, two patients in the pCR group received HAI following systemic chemotherapy after 2012; one of these patients also received anti-PD-1 treatment (**Figure 1**).

### Survival analysis

**Figure 2:**
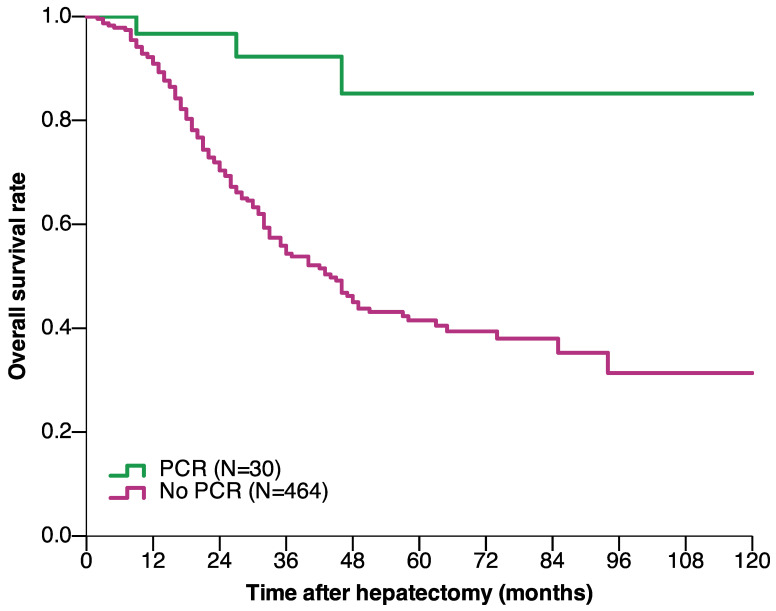
Overall survival of CRLM patients who underwent hepatectomy based on achieving pCR after preoperative chemotherapy (*P* < 0.001) (log-rank test). Abbreviations: CRLM, colorectal liver metastasis; pCR, pathological complete response.

**Figure 3:**
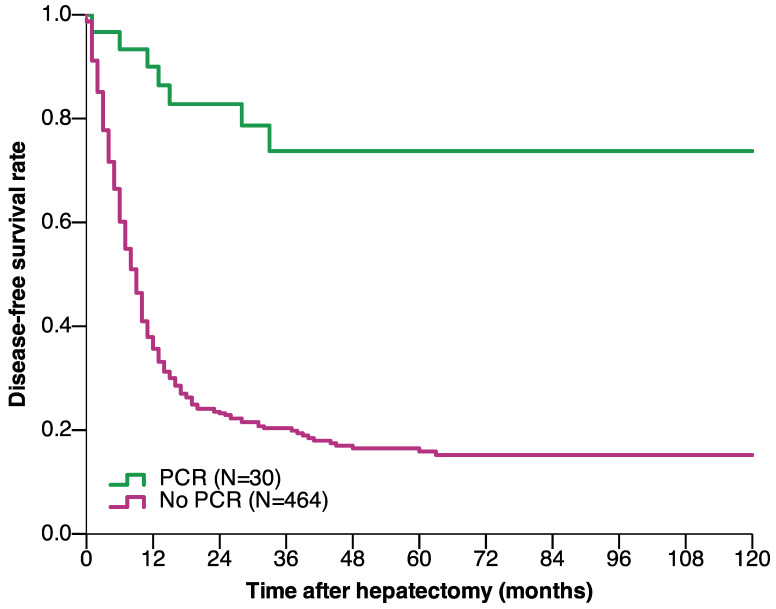
Disease-free survival of CRLM patients who underwent hepatectomy based on achieving pCR after preoperative chemotherapy (*P* < 0.001) (log-rank test). Abbreviations: CRLM, colorectal liver metastasis; pCR, pathological complete response.

The median follow-up duration of the pCR and non-pCR groups were 38 and 24 months, respectively. The OS and DFS were significantly better in patients with pCR than in those without pCR. The 1-, 3-, 5-, and 10-year OS rates in the pCR group were 96.7%, 92.3%, 85.2%, and 85.2%, respectively, and 90.9%, 54.3%, 41.5%, and 31.3%, respectively, in the non-pCR group (*P* < 0.001) (**Figure 2**). The 1-, 3-, 5-, and 10-year DFS rates in the pCR group were 90%, 73.7%, 73.7%, and 73.7%, respectively, and 35.7%, 20.4%, 15.9%, and 15.2%, respectively, in the non-pCR group (*P* < 0.001) (**Figure 3**).

### Predictive factors of pCR

On univariate logistic regression analysis, liver tumor size, preoperative CEA levels, primary T stage, primary tumor location, and irinotecan-based chemotherapy were identified as predictors of pCR following preoperative chemotherapy (**Table 4**). Multivariate analysis identified liver tumor size (<3 vs ≥3 cm, OR [95% CI]: 20.542 [2.738-154.139], *P* = 0.003), preoperative CEA levels (≤20 vs >20 ng/mL, OR [95% CI]: 7.656 [1.005-58.347], *P* = 0.049), primary T stage (T1-2 vs T3-4, OR [95% CI]: 3.131 [1.213-8.082], *P* = 0.018), and primary tumor location (right vs left-sided, OR [95% CI]: 2.808 [1.198-6.580], *P* = 0.017) as independent predictors of pCR after preoperative chemotherapy (**Table 4**).

## Discussion

Chemotherapy response is widely accepted as an important factor affecting the prognosis of patients with CRLM 12. Achievement of pCR suggests that the tumor is adequately responsive to chemotherapy and indicates a very favorable prognosis 8. However, unlike CCR, which is assessed using imaging, pCR can only be confirmed by pathological examination of each tumor. A previous study showed that 41% of metastases that show CCR relapse during follow-up 13, indicating the persistence of cancer cells *in situ*. However, in tumors with pCR, cancer cells are completely replaced by necrotic and fibrotic tissue, and there are no residual cancer cells on pathological examination. However, most cases would still be considered positive based on CT or MRI imaging (**Figure 1E, F**) after preoperative chemotherapy 8. Therefore, the oncological value of pCR is paramount.

In this study, the proportion of pCR in patients receiving preoperative chemotherapy was 6.07%, compared to approximately 4% in previous studies 8, 9. Tanaka et al. reported that 5 of 63 (7.9%) patients achieved pCR after chemotherapy, but the sample was relatively small 13. However, the incidence of pCR in our study was slightly higher than those in earlier studies. The main reason for this phenomenon was the widespread use of intensive preoperative chemotherapy combined with targeted drugs, which cause more pronounced tumor regression 9, 14. More than 30% of the patients in this study received bevacizumab and 20% received cetuximab; this was much higher than the administration rates of previous studies 8, 10, 13. HAI also improves the odds of achieving good pathological response because of higher drug concentrations in the liver 15. In MSI-H CRLM patients, immunotherapy has been considered as a first-line treatment, that can significantly improve the pathological response and prolong patient survival. However, time period analysis shows that the probability of pCR did not significantly increase after 2012 compared with that observed before 2012. Apparently, the pCR rate in CRLM patients has not increased in recent years, despite the development of treatment such as chemotherapy regimens, targeted drugs, HAI, and even immunotherapy. However, since the number of patients with complex and heavy liver metastatic burdens has significantly increased in recent years, the probability of achieving pCR in these cases would be relatively lower than that in patients with low tumor burden receiving traditional chemotherapy regimens. The maintenance of similar pCR rates in recent years also confirmed the importance of advances in effective systemic treatment for the occurrence of pCR.

The long-term prognosis was much better in patients who achieved pCR than in those who did not; patients who achieved pCR had 10-year OS and DFS of 85.2% and 73.7%, respectively, whereas patients who did not achieve pCR had 10-year OS and DFS of 31.3% and 15.2%, respectively. A previous study found that the 10-year OS of patients who achieved pCR reached 68% 8. According to a previous study on the definition of cure in CRLM patients 16, most patients who achieved pCR and experienced no recurrence were cured by surgery. The improved long-term prognosis of patients who experienced pCR in this study may have been related to increased understanding of the biological behavior of CRLM and improved systemic treatment for CRLM in recent years 17. Additionally, with the improvement in surgical techniques and local ablative treatment (LAT), more patients with extrahepatic metastases or liver-limited recurrence undergo extended radical surgery 18-20.

Although pCR is a strong predictor for survival, assessing pCR in a non-invasive manner before surgery remains difficult. Therefore, it would be considerably useful to be able to preoperatively identify CRLM patients who are likely to achieve pCR. In this study, we found that patients with tumor size <3 cm, preoperative CEA <20 ng/mL, earlier T stage, and right-sided primary tumor were more likely to develop pCR after chemotherapy. Auer et al. also found similar correlations between small tumor size, low CEA levels, and pCR 6. The results suggest that patients with smaller tumor burdens after chemotherapy are more likely to achieve pCR. However, this does not imply that the patient will definitely achieve pCR if they demonstrate these features. These factors are also well-known general indicators for a favorable prognosis in patients with CRLM. Adam et al. found that the probability of pCR in patients demonstrating all of these characteristics is only 30% 8. Therefore, we could only conclude that patients with the mentioned factors have a higher likelihood of achieving pCR but are not specific factors for pCR. Another factor found to be closely related to pCR was right-sided primary tumor location. This is interesting since the presence of a right-sided primary is generally considered to be a poor prognotic factor in metastatic colorectal cancer; however, research on the impact of primary tumor location on pCR is lacking. It is known that the molecular biological characteristics of left and right sided colon cancer are different. Right-sided tumors are more likely to demonstrate MSI-H, BRAF mutations, POLE mutations, RAS mutations, and CpG island methylation, compared with left-sided tumors. Immunotherapy has been shown to significantly increase the pathological response rate of metastatic colorectal cancer patients with MSI-H 21. Whether it is possible for certain right-sided tumors with special types of mutations to show higher pathologic response rates needs further studies.

For those patients with resectable liver metastases who demonstrate the above-mentioned characteristics, there is still no evidence that radical surgery is not necessary. Surgery allows us to determine whether residual tumor cells are present on pathological examination; it also aids the identification of new lesions that were not detected on preoperative imaging. This may therefore allow pursuance of the possibility of cure in these patients. However, for patients with a probability of pCR who need extremely complicated liver surgery, several liver lesions can be selected for biopsy or resection to determine the current pathological response status 22. If cancer cells are not found in any of the tumor samples, radical resection may not be needed to remove the remaining liver metastases. It would therefore be possible to achieve long-term survival similar to that of LAT, by administering continuous intensive chemotherapy. Several case reports have described patients with more than 10 unresectable lesions, who showed durable complete responses after systematic chemotherapy 22, 23.

The clinical characteristics described in this study are few of the manifestations of CRLM patients who showed pCR. An underlying molecular biology mechanism is expected to be responsible for the phenomena. Indeed, new methods are emerging for the prediction of pCR, which particularly include radiographic and liquid biopsy techniques. Diffusion-weighted and gadoxetic acid-enhanced MRI provides considerably greater functional imaging information, that reflects the metabolism of tumor cells. In recent years, some studies have used quantitative MRI data and deep learning methods to establish models to identify pCR in CRLM after preoperative chemotherapy 24, 25. The development of liquid biopsy has made it possible to detect ctDNA released in the blood by tumor tissues. The chemotherapy response could also be determined by quantifying ctDNA changes before and after chemotherapy using next-generation sequencing 26. In our center, patients' clinical characteristics, gadoxetic acid-enhanced MRI and contrast-enhanced ultrasonography, tumor markers (CEA and CA19-9), and ctDNA testing results are combined to comprehensively evaluate and identify patients who have a possibility of achieving pCR. In the future, more precise molecular methods are needed to help doctors predict the probability of pCR, this will aid treatment decision making.

There are several limitations in this study. The main disadvantage is the limited sample size with a relatively low incidence of pCR. Therefore, change in a few patients may bias the results, although the percentages were similar to those of previous studies. In addition, a certain degree of heterogeneity in preoperative treatment methods may have also influenced the results; however, the treatment regimen was not found to be an independent factor for pCR. Finally, the indicators obtained in this study reflect that these patients may have good pathological responses to chemotherapy, but they cannot completely determine pCR or rule out the necessity of surgery. These factors need to be validated in a larger sample and new methods for predicting pCR need to be identified in the future.

In conclusion, in the era of targeted drugs and systemic treatment, the proportion of pCR after preoperative chemotherapy in CRLM is approximately 6%. The long-term prognosis of patients who achieve pCR is extremely favorable, and most of them may be cured by surgery. Patients with limited preoperative tumor burdens are more likely to benefit from chemotherapy and achieve pCR.

## Figures and Tables

**Table 1:** Baseline characteristics

Variables	pCR group (n = 30)	Non-pCR group (n = 464)	*P*
**Gender**			0.214
Female	13 (43.3)	150 (32.3)	
Male	17 (56.7)	314 (67.7)	
**Age**			
<65 years	24 (80.0)	364 (78.4)	0.841
≥65 years	6 (20.0)	100 (21.6)	
**Primary tumor**			
T category			**0.034**
T1-2	8 (26.7)	60 (12.9)	
T3-4	22 (73.3)	404 (87.1)	
**N category**			0.294
N0	12 (40.0)	143 (30.8)	
N1-2	18 (60.0)	321 (69.2)	
**Tumor location**			**0.008**
Left-sided	19 (63.3)	384 (82.8)	
Right-sided	11 (36.7)	80 (17.2)	
**Liver metastases**			
Presentation timing			0.936
Metachronous	9 (30.0)	136 (29.3)	
Synchronous	21 (70.0)	328 (70.7)	
**Size**			**<0.001**
<3 cm	29 (96.7)	230 (49.6)	
≥3 cm	1 (3.3)	234 (50.4)	
**Tumor number**			0.671
Single	8 (26.7)	108 (23.3)	
Multiple	22 (73.3)	356 (76.7)	
**RAS status**			0.236
Wild-type	21 (70.0)	274 (59.1)	
Mutated	9 (30.0)	190 (40.9)	
**Preoperative CEA**			**0.001**
≤20 ng/mL	29 (96.7)	321 (69.2)	
>20 ng/mL	1 (3.3)	143 (30.8)	

pCR, pathological complete response; CEA, carcinoembryonic antigen.

**Table 2:** Preoperative chemotherapy details

Variables	pCR group (n = 30)	Non-pCR group (n = 464)	*P*
**Bevacizumab**			0.737
No	21 (70.0)	311 (67.0)	
Yes	9 (30.0)	153 (33.0)	
**Cetuximab**			0.390
No	25 (83.3)	355 (76.5)	
Yes	5 (16.7)	109 (23.5)	
**Oxaliplatin based chemotherapy**			0.119
No	7 (23.3)	174 (37.5)	
Yes	23 (76.7)	290 (62.5)	
**Irinotecan based chemotherapy**			**0.024**
No	24 (80.0)	275 (59.3)	
Yes	6 (20.0)	189 (40.7)	
Chemotherapy cycle (range)	4 (2-10)	4 (1-25)	0.346
**Response to chemotherapy**			0.224
Complete/Partial	17 (56.7)	210 (45.3)	
Stable/Progression	13 (43.3)	254 (54.7)	

pCR, pathological complete response; Response to chemotherapy, tumor response to the last-line chemotherapy.

**Table 3:** Preoperative treatment details of the patients who showed pCR according to the time period

Patients	Before 2012 (n=210)	After 2012 (n=284)	*P*
Total number of pCR cases	13 (6.19%)	17 (6.0%)	0.93
Chemotherapy (Doublet drugs)	9 (69.2%)	6 (35.3%)	0.07
Chemotherapy plus targeted drugs	3 (23.1%)	9 (52.9%)	0.10
HAI	1 (7.7%)	2 (11.8%)	0.81
Immunotherapy	0 (0%)	1 (6%)^ *^	0.92

*One patient received HAI and sequential immunotherapy treatment and was also included in the HAI group. pCR, pathological complete response; HAI, hepatic artery infusion.

**Table 4:** Uni- and multivariable analyses for identifying predictors of pCR

Variables	Univariable			Multivariable		
	OR	95% CI	*P*	OR	95% CI	*P*
Gender (Female vs male)	1.601	0.758-3.382	0.218	-	-	-
Age (<65 vs ≥65 years)	1.099	0.437-2.762	0.841	-	-	-
Primary tumor						
T category(T1-2 vs T3-4)	2.448	1.043-5.748	**0.040**	3.131	1.213-8.082	**0.018**
N category (N0 vs N1-2)	1.497	0.702-3.189	0.296	-	-	-
Location(Right vs left)	2.799	1.273-6.066	**0.010**	2.808	1.198-6.580	**0.017**
Liver metastases						
Presentation time (Metachronous vs synchronous)	0.936	0.462-2.314	0.994	-	-	-
Tumor size (<3 vs ≥3 cm)	29.504	3.986-218.39	**0.001**	20.542	2.738-154.139	**0.003**
Tumor number (Single vs multiple)	1.199	0.519-2.769	0.671	-	-	-
RAS status (Wild-type vs mutated)	1.618	0.725-3.610	0.240	-	-	-
Oxaliplatin based chemotherapy (Yes vs No)	1.971	0.829-4.690	0.125	-	-	-
Irinotecan based chemotherapy (No vs Yes)	2.749	1.103-6.85	**0.030**	2.231	0.856-5.814	0.100
Bevacizumab (No vs Yes)	1.148	0.513-2.566	0.737	-	-	-
Cetuximab (No vs Yes)	1.535	0.574-4.106	0.393	-	-	-
Response to chemotherapy (Stable/Progression vs Complete/Partial)	1.582	0.751-3.331	0.228	-	-	-
Preoperative CEA (≤20 ng/mL vs >20 ng/mL)	12.919	1.743-95.764	**0.012**	7.656	1.005-58.347	**0.049**

pCR, pathological complete response; CEA, carcinoembryonic antigen; Response to chemotherapy, tumor response to the last-line chemotherapy; OR, odds ratio; CI, Confidence Interval.
